# Metagenomes from Eastern Brazilian Amazonian Floodplains in the Wet and Dry Seasons

**DOI:** 10.1128/mra.00432-22

**Published:** 2022-07-19

**Authors:** Andressa M. Venturini, Júlia B. Gontijo, Aline G. da França, José M. S. Moura, Klaus Nüsslein, Brendan J. M. Bohannan, Jorge L. M. Rodrigues, Siu M. Tsai

**Affiliations:** a Cell and Molecular Biology Laboratory, Center for Nuclear Energy in Agriculture, University of São Paulo, Piracicaba, SP, Brazil; b Princeton Institute for International and Regional Studies, Princeton University, Princeton, New Jersey, USA; c Center for Interdisciplinary Formation, Federal University of Western Pará, Santarém, PA, Brazil; d Department of Microbiology, University of Massachusetts, Amherst, Massachusetts, USA; e Institute of Ecology and Evolution, University of Oregon, Eugene, Oregon, USA; f Department of Land, Air, and Water Resources, University of California - Davis, Davis, California, USA; University of Southern California

## Abstract

Here, we report the metagenomes from two Amazonian floodplain sediments in eastern Brazil. Tropical wetlands are well known for their role in the global carbon cycle. Microbial information on this diversified and dynamic landscape will provide further insights into its significance in regional and global biogeochemical cycles.

## ANNOUNCEMENT

Floodplains and wetlands constitute 14% of the total area of the Amazon basin (1) and are considered the largest natural geographic source of methane (CH_4_) in the tropics (2). Therefore, several studies have investigated the CH_4_-producing and -consuming microbial communities in these sediments and their responses to a range of environmental factors using 16S rRNA amplicon sequencing (3–5). However, their overall microbial taxonomic and functional diversity remains little explored. Here, we report 12 metagenomes from two Amazonian floodplains in the wet and dry seasons.

The samplings were carried out in two floodplains in the State of Pará, Brazil, namely, one located at the Amazon River (FP2, “Maicá”, 2°28′11.2″S 54°38′49.9″W) and the other at the intersection between the Amazon and the Tapajós rivers (FP3, “Açu”, 2°22′44.8″S 54°44′21.1″W). The Amazon and Tapajós are considered whitewater and clearwater rivers, respectively, according to Junk et al. (6). Sediment samples from a depth of 0 to 10 cm were collected using a corer (5-cm diameter by 10-cm depth) at both sites in the wet and dry seasons (May and October 2016, respectively) in triplicate, totaling 12 samples, and homogenized thoroughly. Total DNA was extracted in duplicate from 0.25 g of sediment using the PowerLyzer PowerSoil DNA Isolation Kit (Qiagen, Hilden, Germany), following an optimized protocol for Amazonian sediments (7). Metagenomic libraries were constructed using the NEBNext Ultra II DNA Library Prep Kit for Illumina (New England BioLabs, Inc., Ipswich, MA) and paired-end sequenced (2 × 150 bp) on an Illumina HiSeq 2500 instrument (Illumina, Inc., San Diego, CA) at Novogene Co., Ltd. (Beijing, China). Detailed information about the study sites, sampling, sediment physicochemical properties, and DNA extraction and quantification have been described previously (5).

Metagenomic reads were imported into the KBase platform (8), and default parameters were used for all software unless otherwise specified. Reads were evaluated using FastQC v0.11.9 (9), trimmed and filtered using Trimmomatic v0.36 (adapters, TruSeq3-PE-2; seed mismatches, 5; sliding window size, 5; sliding window minimum quality, 20; head crop length, 10; leading minimum quality, 20; trailing minimum quality, 20; minimum read length, 70) (10), and again evaluated using FastQC v0.11.9 (9). Overlapping paired-end reads were joined with FASTQ-JOIN v2.0.2 (8, 11) and taxonomically classified using Kaiju v1.7.3 (taxonomic level, phylum/class; reference database, NCBI BLAST nr+euk; low abundance filter, 0.01%; subsample percent, 100%) (12). The results were plotted using ggplot2 3.3.5 (13) in R 4.1.2 (14).

**FIG 1:**
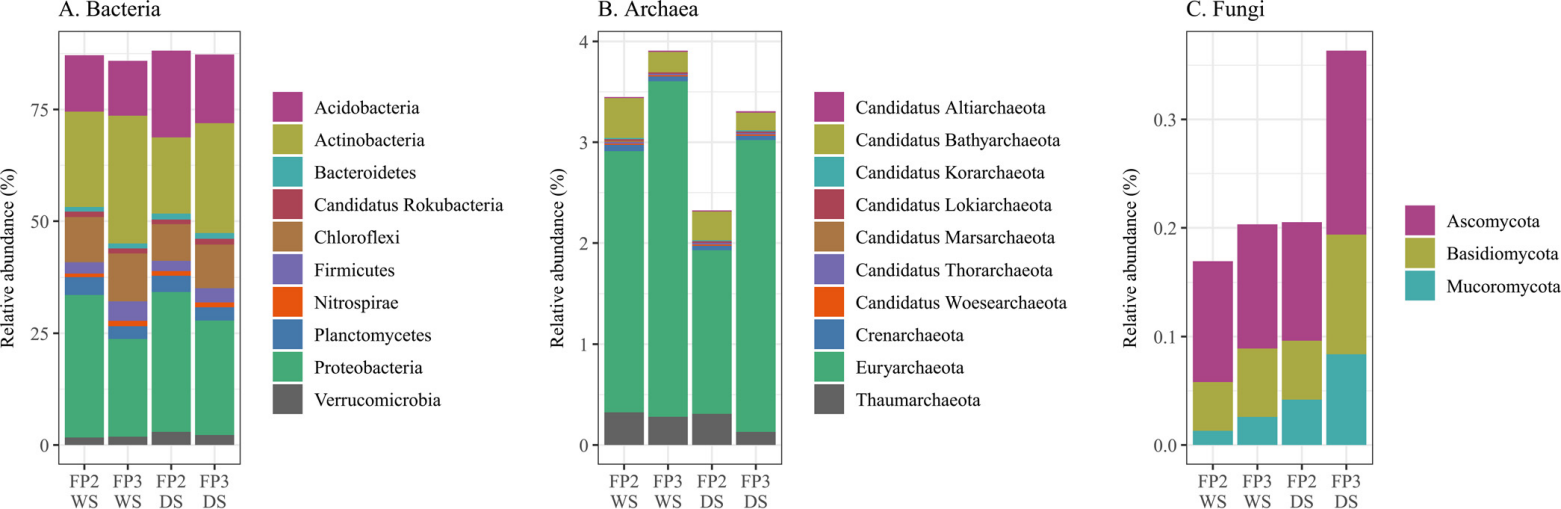
Taxonomic classification of the sequence reads at the phylum level. (A) Most abundant bacterial phyla (mean relative abundance of > 1% across samples). (B) Archaeal phyla. (C) Fungal phyla. Relative abundance calculated based on the classified reads. WS, wet season; DS, dry season.

The metagenomic samples had between 22 and 31 million 150-bp long paired-end reads (Table 1). After quality control, between 19 and 29 million paired-end reads remained, ranging from 70 to 140 bp. The joining of the overlapping paired-end reads resulted in samples with between 9 and 15 million reads, ranging from 76 to 274 bp. A considerable part of the reads (mean of 42% across samples) was not classified. Most of the classified reads were assigned to *Bacteria*, but also *Archaea*, *Fungi*, and viruses (Fig. 1). The most dominant phyla (mean relative abundance of > 10% across samples), among the 90 microbial phyla found, were *Proteobacteria*, *Actinobacteria*, and *Acidobacteria*.

**TABLE 1:** Results of 12 metagenomic samples

Sample	Site	Season	Sediment depth (cm)	Raw sequences		Cleaned sequences		Joined sequences		BioSample no.	SRA no.
				No. of paired-end sequences	Length (bp)	No. of paired-end sequences	Length (bp)	No. of sequences	Length (bp)		
M1	FP2	Wet	0–10	31,488,501	150	29,364,157	70–140	10,771,256	80–274	SAMN28058191	SRR19084119
M2	FP2	Wet	0–10	29,116,938	150	27,014,226	70–140	12,575,705	80–274	SAMN28058192	SRR19084118
M3	FP2	Wet	0–10	27,241,040	150	25,337,441	70–140	9,974,807	84–274	SAMN28058193	SRR19084115
M4	FP3	Wet	0–10	26,476,619	150	24,083,103	70–140	9,194,677	76–274	SAMN28058194	SRR19084114
M5	FP3	Wet	0–10	22,244,668	150	19,225,440	70–140	9,616,174	81–274	SAMN28058195	SRR19084113
M6	FP3	Wet	0–10	27,177,687	150	24,535,240	70–140	11,683,630	84–274	SAMN28058196	SRR19084112
M7	FP2	Dry	0–10	25,506,167	150	21,673,391	70–140	10,228,313	83–274	SAMN28058197	SRR19084111
M8	FP2	Dry	0–10	25,273,528	150	21,972,821	70–140	11,907,897	83–274	SAMN28058198	SRR19084110
M9	FP2	Dry	0–10	27,627,625	150	25,293,872	70–140	14,575,816	81–274	SAMN28058199	SRR19084109
M10	FP3	Dry	0–10	27,345,380	150	24,609,088	70–140	11,042,678	86–274	SAMN28058200	SRR19084108
M11	FP3	Dry	0–10	29,299,442	150	26,571,413	70–140	10,860,484	76–274	SAMN28058201	SRR19084117
M12	FP3	Dry	0–10	24,643,128	150	22,183,799	70–140	9,214,968	78–274	SAMN28058202	SRR19084116

### Data availability.

The raw metagenomic sequences are available in the NCBI Sequence Read Archive (SRA) under the umbrella project PRJNA782633. The raw sequences, apps, and all the outputs of the analyses described here are also available on the KBase platform at https://www.doi.org/10.25982/113717.182/1864845.
